# Systemic lupus erythematosus is causally associated with hypothyroidism, but not hyperthyroidism: A Mendelian randomization study

**DOI:** 10.3389/fimmu.2023.1125415

**Published:** 2023-02-13

**Authors:** Qiong Qin, Ling Zhao, Ao Ren, Wei Li, Ruidong Ma, Qiufeng Peng, Shiqiao Luo

**Affiliations:** Department of Hepatobiliary Surgery, The First Affiliated Hospital of Chongqing Medical University, Chongqing, China

**Keywords:** systemic lupus erythematosus, hyperthyroidism, hypothyroidism, Mendelian randomization, GWAS, SNPs

## Abstract

**Background:**

The relationship between systemic lupus erythematosus (SLE) and thyroid diseases is still controversial. Due to confounders and reverse causation, previous studies were not convincing. We aimed to investigate the relationship between SLE and hyperthyroidism or hypothyroidism by Mendelian randomization (MR) analysis.

**Methods:**

We performed a two-step analysis using bidirectional two-sample univariable and multivariable MR (MVMR) to explore the causality of SLE and hyperthyroidism or hypothyroidism in three genome-wide association studies (GWAS) datasets, including 402,195 samples and 39,831,813 single-nucleotide polymorphisms (SNPs). In the first step analysis, with SLE as exposure and thyroid diseases as outcomes, 38 and 37 independent SNPs strongly (*P* < 5*10^-8^) associated with SLE on hyperthyroidism or SLE on hypothyroidism were extracted as valid instrumental variables (IVs). In the second step analysis, with thyroid diseases as exposures and SLE as outcome, 5 and 37 independent SNPs strongly associated with hyperthyroidism on SLE or hypothyroidism on SLE were extracted as valid IVs. In addition, MVMR analysis was performed in the second step analysis to eliminate the interference of SNPs that were strongly associated with both hyperthyroidism and hypothyroidism. 2 and 35 valid IVs for hyperthyroidism on SLE and hypothyroidism on SLE were obtained in MVMR analysis. MR results of two steps analysis were estimated respectively by multiplicative random effects-inverse variance weighted (MRE-IVW), simple mode (SM), weighted median (WME) and MR-Egger regression methods. Sensitivity analysis and visualization of MR results were performed by heterogeneity test, pleiotropy test, leave-one-out test, scatter plots, forest plots and funnel plots.

**Results:**

The MRE-IVW method in the first step of MR analysis revealed that SLE was causally associated with hypothyroidism (OR = 1.049, 95% CI = 1.020-1.079, *P* < 0.001), but not causally associated with hyperthyroidism (OR = 1.045, 95% CI = 0.987-1.107, *P* = 0.130). In the inverse MR analysis, the MRE-IVW method revealed that both hyperthyroidism (OR = 1.920, 95% CI = 1.310-2.814, *P* < 0.001) and hypothyroidism (OR = 1.630, 95% CI = 1.125-2.362, *P* = 0.010) were causally associated with SLE. Results from other MR methods were consistent with MRE-IVW. However, when MVMR analysis was performed, there was no longer a causal relationship of hyperthyroidism on SLE (OR = 1.395, 95% CI = 0.984-1.978, *P* = 0.061), nor was there a causal relationship of hypothyroidism on SLE (OR = 1.290, 95% CI = 0.823-2.022, *P* = 0.266). The stability and reliability of the results were confirmed by sensitivity analysis and visualization.

**Conclusions:**

Our univariable and multivariable MR analysis revealed that systemic lupus erythematosus was causally associated with hypothyroidism, but did not provided evidence to support a causal relationship of hypothyroidism on SLE or between SLE and hyperthyroidism.

## Introduction

Systemic lupus erythematosus (SLE) is an autoimmune disease characterized by increased anti-nuclear antibodies, anti-dsDNA antibodies, anti-Sm antibodies and antiphospholipid antibodies (1). The global prevalence and mortality rates of SLE range from 13/100,000 to 7713.5/100,000 and from 0.01/100,000 to 2.71/100,000, respectively (2). Both the highest prevalence and highest mortality rates are in Africa, where up to 44% of SLE patients die each year (2). Overall, the prevalence and mortality have shown an increasing trend. SLE is more common in young women and the highest prevalence in women is 8 times higher than in men (1). Compared with men, female SLE patients have an earlier peak age of onset (30-50 years *vs*. 50-70 years) and a higher risk of death, which are more pronounced in black people. SLE can severely and extensively affect human body, including skin mucosa, heart, kidney, skeletal muscle, respiratory system, blood system, digestive system and nervous system (3). In addition, SLE can coexist with hyperthyroidism, hypothyroidism and other autoimmune diseases.

Hyperthyroidism and hypothyroidism are common endocrine diseases. The former produces more thyroid hormones, while the latter does the opposite. The causes of hyperthyroidism include Graves’ disease (GD), thyroiditis, drugs, HCG and pituitary TSH-omas, of which GD is the most common and accounts for 80% (4). GD is an autoimmune disease with elevated thyrotropin receptor antibodies (TRAb) (5). Hypothyroidism can be divided into thyroid hormone resistance syndrome, primary and secondary hypothyroidism, of which primary hypothyroidism accounts for 95% (6). Primary hypothyroidism is mainly caused by immune abnormalities, thyroidectomy and overtreatment of hyperthyroidism, and elevated thyroid peroxidase antibodies (TPOAb) and thyroglobulin antibodies (TgAb) can be detected in some patients (7). Both hyperthyroidism and hypothyroidism can cause damage to the eight systems of human body, as well as to the skin and eyes (8). Early prevention and treatment of thyroid diseases can reduce these damages.

Clinically, SLE patients coexisting with hyperthyroidism or hypothyroidism are not rare (9, 10) and the sequence of diseases onset is different. Therefore, it is not clear whether SLE causes thyroid diseases, or thyroid diseases cause SLE, or the treatment of the former disease causes the latter disease. Currently, studies on the relationship between SLE and hyperthyroidism or hypothyroidism have not reached a consistent conclusion. Some studies revealed that SLE patients were more likely to develop hyperthyroidism or hypothyroidism than the general population (11–13), but other studies provided no evidence of such a difference (14). Several studies revealed that a genetic overlap existed between SLE and autoimmune thyroid diseases, such as R620W polymorphism of the PTPN22 gene (15). However, these observational studies were vulnerable to confounders and reverse causation. Liu et al. (16) revealed a bidirectional causal relationship between SLE and primary hypothyroidism through Mendelian randomization analysis, but they did not explore the relationship between SLE and hyperthyroidism. In this study, we investigated the causal relationship between SLE and hypothyroidism, and SLE and hyperthyroidism through a two-sample Mendelian randomized analysis, intending to provide convincing evidence for the early prevention of SLE and thyroid diseases.

Mendelian randomization (MR) is a statistical analysis method used to infer epidemiological etiology and follows the Mendelian law of heredity that alleles are randomly assigned. In MR analysis, single-nucleotide polymorphisms (SNPs) are used as instrumental variables (IVs) to explore the potential causal relationship between phenotypes (exposures) and diseases (outcomes). Valid IVs should conform to three basic assumptions: (1) Valid IVs should be strongly associated with exposure and independent (*P* < 5*10^-8^, linkage disequilibrium [LD] *r* ^2^<0.001); (2) Valid IVs should not be associated with confounders that mediate exposure and outcome; (3) Valid IVs cannot be directly related to outcome or affect the outcome except through the exposure of interest (17, 18). Compared with observational studies which are difficult to identify the sequence of onset between exposure and outcome, SNPs are innate and cannot be altered by acquired factors, including exposures, confounders and outcomes. Thus, Mendelian randomization can reveal a true causal relationship between exposure and outcome.

We intend to conduct a bidirectional two-sample univariable and multivariable MR (19) study to investigate whether there is a direct causal relationship between SLE and hyperthyroidism or hypothyroidism, so as to provide theoretical support for clinical practice.

## Methods

### Source of exposure and outcome GWAS data

To avoid bias caused by the overlap of exposure and outcome samples, we obtained exposure and outcome samples from different databases. When selecting exposure and outcome GWAS datasets, European origin of population, more comprehensive disease type, larger size of population samples and SNPs, more comprehensive gender composition, and time of data publication were comprehensively taken into account. The GWAS dataset associated with SLE was established by European Bioinformatics Institute, with the ID number of “ebi-a-GCST003156”, and was comprised of 14,267 samples and 7,071,163 SNPs (https://gwas.mrcieu.ac.uk/). The GWAS datasets associated with thyroid diseases were established by FinnGen Biobank. The hyperthyroidism GWAS dataset was comprised of 173,938 samples and 16,380,189 SNPs with the ID numbers of “finn-b-AUTOIMMUNE_HYPERTHYROIDISM” and the hypothyroidism GWAS dataset was comprised of 213,990 samples and 16,380,461 SNPs, with the ID number of “finn-b-E4_HYTHYNAS” (https://gwas.mrcieu.ac.uk/). Details of the three GWAS datasets were listed in **Supplementary Table 1**.

### Ethics statement

All GWAS datasets in this study obtained from European Bioinformatics Institute and FinnGen Biobank are publicly published and have received corresponding ethical approval. Separate ethical approval was not required for this study.

### Mendelian randomization analysis

**Figure 1:**
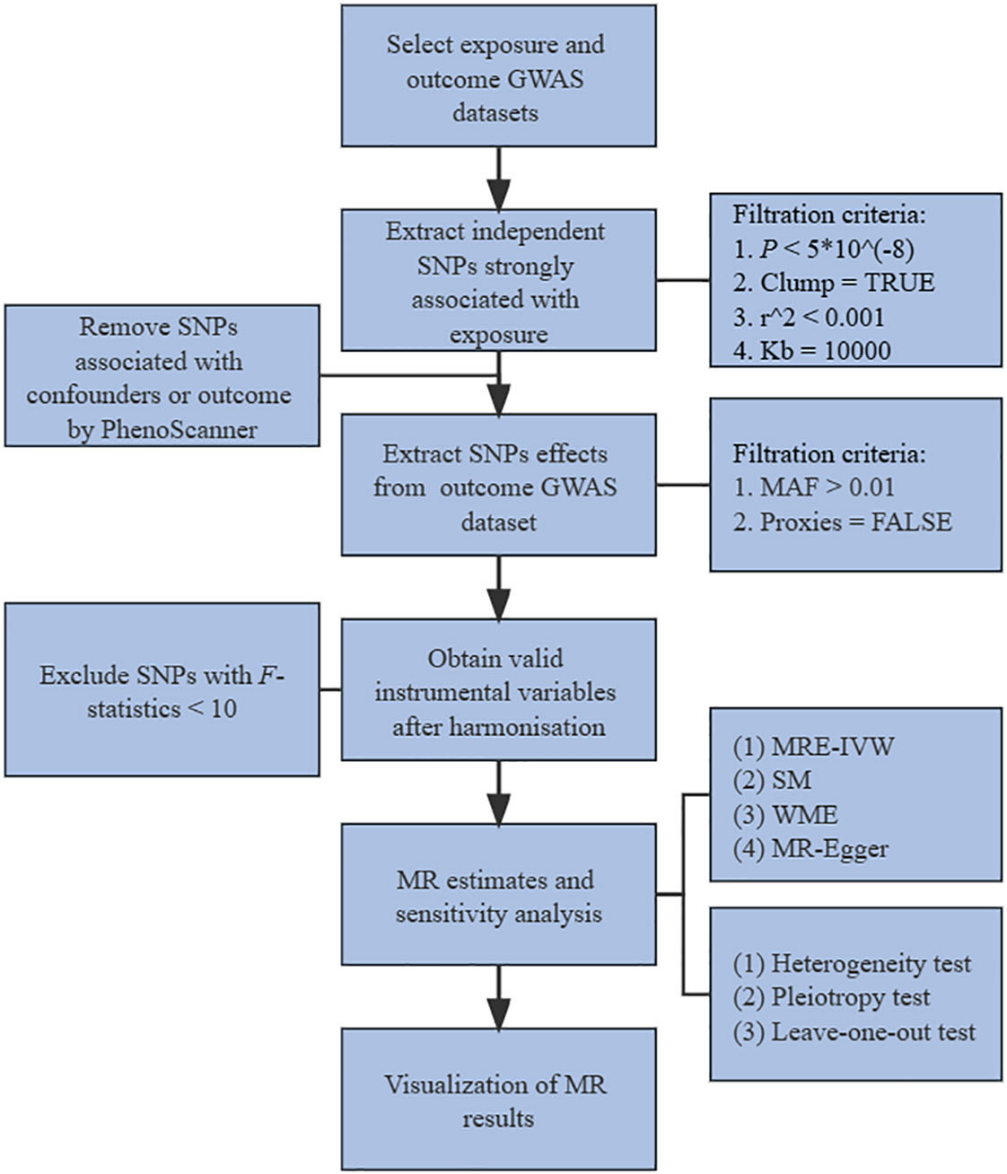
Flow chart of study. GWAS, genome-wide association studies. SNPs, single-nucleotide polymorphisms. MAF, minor allele frequency. MR, mendelian randomization. MRE-IVW, multiplicative random effects-inverse variance weighted. SM, simple mode. WME, weighted median.

In our study, two-step of two-sample MR analysis was performed, including bidirectional univariable and multivariable analysis. MR analysis in this study of each step was achieved mainly through the following operations: Firstly, extracted independent SNPs strongly correlated with exposure with filtration criteria of *P* < 5*10^-8^ in 10000kb window around a leader SNP with low association with other SNPs in this region (LD, *r* ^2^ < 0.001). Then searched these SNPs in PhenoScanner (www.phenoscanner.medschl.cam.ac.uk), and removed SNPs associated with confounders and outcomes. Confounders mainly referred to the potential risk factors other than exposure of interest that could cause the outcome. Common risk factors for SLE included but were not limited to smoking, alcohol consumption, obesity and vitamin D deficiency (20). Common risk factors for hyperthyroidism and hypothyroidism included but were not limited to smoking, iodine consumption, ethnicity, immunosuppressants, endocrine disruptors (21). Secondly, extract SNPs effects from outcome GWAS dataset with filtration criteria of MAF > 0.01. Next, obtained valid IVs after harmonizing exposure and outcome effects and removing SNPs with *F*-statistics < 10 or with failure to harmonize, including containing incompatible alleles and being palindromic with intermediate allele frequencies. Then, the MR evaluation of valid IVs was carried out by multiplicative random effects-inverse variance weighted (MRE-IVW), simple mode (SM), weighted median (WME) and MR-Egger regression methods. Finally, conducted sensitivity analysis and visualization of the MR results. The flow chart of study was shown in **Figure 1**.

In the first step of analysis, SLE was taken as exposure, hyperthyroidism or hypothyroidism was taken as outcome, respectively, and only two-sample univariable MR analysis was used. 45 SNPs with LD strongly correlated with SLE were extracted from SLE GWAS dataset. SNPs of rs389884, rs597808 and rs6679677 were removed for being associated with confounders or hyperthyroidism by searching in PhenoScanner. 41 SNPs effects were extracted from hyperthyroidism and three of them were removed for failure to harmonize, including rs28361029, rs2736332 and rs28834423 (**Supplementary Table 2**). Similarly, 4 SNPs of rs4274624, rs597808, rs6679677 and rs7899626 were removed for being associated with confounders or hypothyroidism from 45 SNPs strongly associated with SLE. Three SNPs of rs28361029, rs2736332 and rs28834423 were removed for harmonize failure from 40 SNPs effects extracted from hypothyroidism (**Supplementary Table 3**).

In the second step of analysis, thyroid diseases were taken as exposures, SLE was taken as outcome. 7 and 52 SNPs strongly correlated were extracted from hyperthyroidism and hypothyroidism GWAS datasets, respectively. rs7310615 was associated with confounders and removed from the 52 SNPs. Among 6 SNPs effects extracted from SLE GWAS for the relationship of hyperthyroidism on SLE, rs9265890 was removed for failure to harmonize (**Supplementary Table 4**). 7 SNPs were failed to harmonize and removed from 44 SNPs effects extracted from SLE GWAS for the relationship of hypothyroidism on SLE, including rs3132487, rs13289602, rs1993945, rs2983511, rs707937, rs7754251 and rs932036 (**Supplementary Table 5**). However, in univariable MR analysis, we observed that some SNPs were strongly associated with both hyperthyroidism and hypothyroidism. Since it was not really clear at the beginning of the study whether there was a true causal relationship between hyperthyroidism or hypothyroidism and SLE, the univariable MR analysis did not remove these SNPs as it was so strongly associated with exposure of interest but it could not yet be identified as confounders. But given the hypothesis that hyperthyroidism and hypothyroidism might act as confounders to each other and bring interference to the results of univariable MR analysis. We further performed multivariable MR analysis. 45 SNPs strongly associated with hyperthyroidism or hypothyroidism were extracted from two GWAS datasets. rs7310615 was removed for being associated with confounders and rs7754251 was removed for being palindromic with intermediate allele frequencies. 41 SNPs effects were extracted from SLE GWAS, of which rs13289602, rs1993945, rs2983511, rs707937 and rs932036 were removed for failure of harmonize. Finally, 2 valid IVs of rs179247 and rs6679677 were used for MR estimate of hyperthyroidism on SLE, and 35 valid IVs — only rs179247 was removed — were used for MR estimate of hypothyroidism on SLE (**Supplementary Table 6**).

### Statistical analysis

All statistical analyses for this study were performed using R v4.0.5 and the TwoSampleMR package. The *F*-statistic was used in the estimate of valid IVs from harmonisation. Currently, there were several commonly used formulas for calculation of *F*-statistics, such as *F* = (N-k-1)/k×R^2^/(1-R^2^) or *F* = β^2^/se^2^. The formula used in this study was β^2^/se^2^ (β, effect size(exposure); se, standard error(exposure)), referring to the univariable and multivariable MR analysis study of Chen et al. (22). Only valid IVs with *F*-statistic > 10 were defined as high intensity and would be retained for MR estimates, sensitivity analysis and visualization. In the sensitivity analysis, heterogeneity test, pleiotropy test and leave-one-out test were used to verify the stability and reliability. We evaluated the heterogeneity by using Cochran’s *Q* statistics and *I^2^* statistics (23). If the heterogeneity existed (*P* < 0.05), the causal relationship should be consistent with the results estimated by MRE-IVW (24). In the pleiotropy test, intercepts calculated by MR-Egger regression were used to assess the horizontal pleiotropy of valid IVs. The more the intercepts deviated from zero, the greater the probability of horizontal pleiotropy existence (*P* < 0.05) (25). In the leave-one-out test, after removing a certain valid IV one by one, the MRE-IVW method was used to calculate the results to identify whether the removed valid IV could significantly change the MR results. If the distribution of “ALL” line was consistent with the results of MR estimates, it indicated that the MR results were stable and reliable (26). In our study, scatter plots, forest plots and funnel plots were used for the presentation of MR results.

## Results

### Univariable MR estimates

In the first step analysis, a total of 38 and 37 valid IVs strongly associated with SLE on hyperthyroidism and SLE on hypothyroidism were included into MR estimates, respectively (**Supplementary Tables 2, 3**). The evaluation of 38 valid IVs effects for SLE on hyperthyroidism showed the consistent results that SLE was not causally associated with hyperthyroidism. *P* values of MRE-IVW, SM, WME and MR-Egger were more than 0.05 (**Table 1**). However, MRE-IVW evaluation of 37 valid IVs effects for SLE on hypothyroidism provided evidence to support that causal relationship existed (OR = 1.049, 95% CI = 1.020-1.079, *P* < 0.001), and results estimated by other methods were consistent with MRE-IVW (**Table 1**).

**Table 1:** Bidirectional Two-sample univariable Mendelian randomization analysis of the relationship between SLE and hyperthyroidism or hypothyroidism.

Exposure	Outcome	Method	nSNP	OR	95%CI	*P*-value
SLE	Hyperthyroidism	MRE-IVW	38	1.045	(0.987, 1.107)	0.130
		SM		1.040	(0.950, 1.140)	0.397
		WME		1.025	(0.935, 1.124)	0.593
		MR-Egger		1.134	(0.994, 1.294)	0.070
SLE	Hypothyroidism	MRE-IVW	37	1.049	(1.020, 1.079)	<0.001
		SM		1.052	(1.024, 1.079)	<0.001
		WME		1.031	(1.007, 1.055)	0.011
		MR-Egger		1.075	(1.011, 1.142)	0.027
Hyperthyroidism	SLE	MRE-IVW	5	1.920	(1.310, 2.814)	<0.001
		SM		2.049	(1.652, 2.542)	<0.001
		WME		1.254	(0.994, 1.582)	0.056
		MR-Egger		3.165	(1.373, 7.298)	0.074
Hypothyroidism	SLE	MRE-IVW	37	1.630	(1.125, 2.362)	0.010
		SM		1.484	(1.189, 1.851)	<0.001
		WME		1.417	(1.153, 1.741)	<0.001
		MR-Egger		1.920	(0.751, 4.908)	0.182

SLE, systemic lupus erythematosus; SNP, single-nucleotide polymorphism; MRE-IVW, multiplicative random effects-inverse variance weighted; SM, simple mode; WME, weighted median.

In the second step of inverse analysis, a total of 5 and 37 valid IVs associated with hyperthyroidism on SLE and hypothyroidism on SLE were obtained after harmonisation (**Supplementary Tables 4, 5**). Evaluation estimated by MRE-IVW (*P* < 0.001) and SM (*P* < 0.001) methods revealed the existence of causality between hyperthyroidism and SLE, but WME and MR-Egger methods did not (*P* = 0.056 and 0.074, respectively, **Table 1**). Estimates from MRE-IVW (*P* = 0.010), SM (*P* < 0.001) and WME (*P* < 0.001) methods revealed that hypothyroidism is causally associated with SLE. But result from MR-Egger regression method did not identify this causal relationship (*P* = 0.182).

### Multivariable MR estimates

In univariable MR analysis, while revealing the causal relationship between hyperthyroidism or hypothyroidism and SLE, we also found that after removing the SNPs associated with confounders, there were still some SNPs that were strongly associated not only with hyperthyroidism, but also with hypothyroidism. Therefore, multivariable analysis was performed to verify whether causality still existed after the removal of these SNPs. A total of 36 valid IVs were retained for MR estimate, 2 SNPs were included in the analysis of hyperthyroidism on SLE and 35 were included in the analysis of hypothyroidism on SLE (**Supplementary Table 6**). The multivariable analysis revealed no causal relationship existed in hyperthyroidism on SLE (OR = 1.395, 95% CI = 0.984-1.978, *P* = 0.061, **Table 2**), nor existed in hypothyroidism on SLE (OR = 1.290, 95% CI = 0.823-2.022, *P* = 0.266, **Table 2**).

**Table 2:** Two-sample multivariable Mendelian randomization analysis of the relationship for hyperthyroidism or hypothyroidism on SLE.

Exposure	Outcome	Method	nSNP	OR	95%CI	P-value
Hyperthyroidism	SLE	MRE-IVW	2	1.395	(0.984, 1.978)	0.061
Hypothyroidism	SLE	MRE-IVW	35	1.290	(0.823, 2.022)	0.266

SLE, systemic lupus erythematosus; SNP, single-nucleotide polymorphism; MRE-IVW, multiplicative random effects-inverse variance weighted.

### *F*-statistics and sensitivity analysis

*F*-statistics were calculated for each valid IVs, none was less than 10 (**Supplementary Tables 2–6**). In sensitivity analysis, MRE-IVW and MR-Egger regression methods were used in heterogeneity test to calculate Cochran’s *Q* statistics (**Supplementary Table 7**). No heterogeneity was found in valid IVs used to estimated effect of SLE on hyperthyroidism (*I^2^* = 0, *P* > 0.05, **Supplementary Table 7**). Although heterogeneities existed in valid IVs used to estimated effect of SLE on hypothyroidism, hyperthyroidism on SLE and hypothyroidism on SLE, results estimated by MRE-IVW still kept stable. In our pleiotropy test, no horizontal pleiotropy was found in the valid IVs (**Supplementary Table 8**). In leave-one-out test, although the removal of rs9275576 changed the effect of hyperthyroidism on SLE (*P* = 0.190, **Supplementary Table 9C**), and the removal of rs6679677 changed the effect of hypothyroidism on SLE (*P* = 0.052, **Supplementary Table 9D**), all effects were not changed and “ALL” lines in the leave-one-out test plots were still greater than 0 (**Supplementary Figures 1C, D**), which indicated that our study had sufficient statistical power and the MR results stood up to the test of sensitivity analysis.

### Visualization of MR

**Figure 2:**
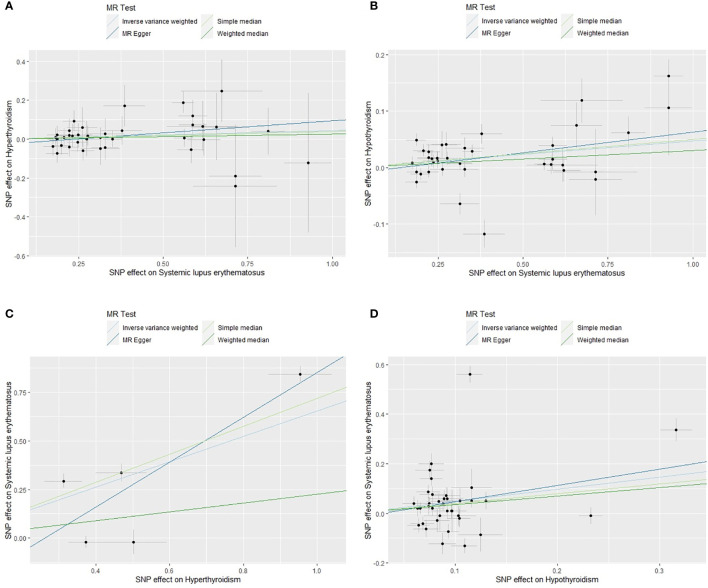
Scatter plots of causal effect estimates for SLE on hyperthyroidism **(A)**, SLE on hypothyroidism **(B)**, hyperthyroidism on SLE **(C)**, hypothyroidism on SLE **(D)**, with all 38, 37, 5 and 37 valid instrumental variables. SLE, systemic lupus erythematosus.

**Figure 3:**
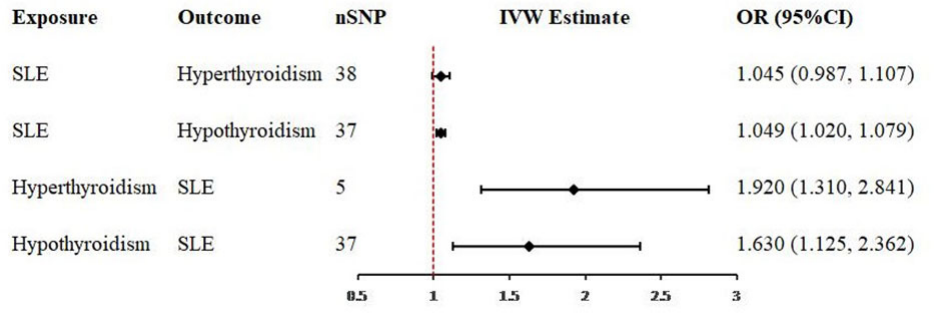
Forest plots of causal effect estimates in univariable Mendelian analysis. SLE, systemic lupus erythematosus; SNP, single-nucleotide polymorphism; IVW, inverse variance weighted.

**Figure 4:**
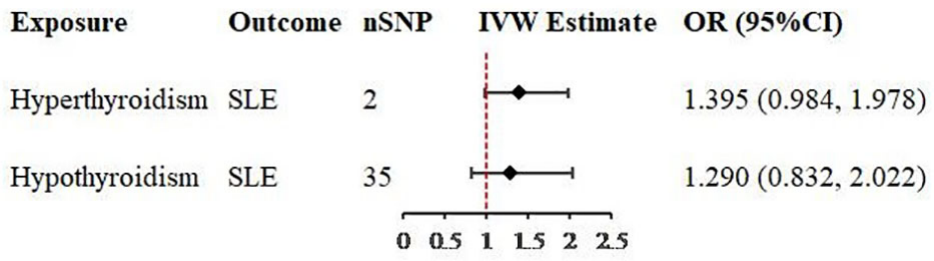
Forest plots of causal effect estimates in multivariable Mendelian analysis. SLE, systemic lupus erythematosus; SNP, single-nucleotide polymorphism; IVW, inverse variance weighted.

The scatter plots of valid IVs causal effects estimate for SLE on hyperthyroidism or hypothyroidism and hyperthyroidism or hypothyroidism on SLE were shown in **Figure 2**. Although the scatter plot of causal effect for SLE on hyperthyroidism seemed to present a tendency of positive correlation (**Figure 2A**), forest plot did not present a causal relationship and was consistent with MR results (**Figure 3**). The other three scatter plots and forest plots all presented a clear causal relationship (**Figures 2B–D**, **3**). For each valid IVs, the forest plots were presented in **Supplementary Figure 3**. Except for several outliers, valid IVs estimated by MRE-IVW in univariable MR analysis were distributed symmetrically in the funnel plots (**Supplementary Figure 2**), which indicated the SNPs in this study followed the random distribution rule. However, in multivariate analysis, the causal relationship between hyperthyroidism or hypothyroidism and SLE no longer existed, as shown in the forest plots (**Figure 4**).

## Discussion

Previous studies found that there were not only nuclear and cytoplasmic antigen antibodies in the serum of SLE patients, but also some common antibodies of other autoimmune diseases, such as anti-phospholipid antibodies, anti-Ro antibodies, anti-La antibodies, etc. (27) The GWAS studies found that some SNPs associated with SLE were also associated with multiple autoimmune diseases (28). Liu et al. (16) revealed a bidirectional causal relationship between SLE and primary hypothyroidism through bidirectional univariable Mendelian randomization analysis, but they did not explore the relationship between SLE and hyperthyroidism and did not perform multivariable MR analysis. In clinical practice, SLE patients coexisting with hyperthyroidism were also common and the sequence of SLE and thyroid diseases onset was not clear for some patients. Wang (29) and Tsuji et al. (30). found that HLA-B, HLA-D8, HLA-DR3, HLA-DR13, HLA-DR16 and HLA-DRW3 were positively associated with an increased risk of SLE. Among these markers, HLA-B and HLA-DRW3 had been confirmed to be positively associated with hyperthyroidism (31), and HLA-D8 and HLA-DR3 had been confirmed to be positively associated with hypothyroidism (32). Based on these findings, the relationship between SLE and hyperthyroidism or hypothyroidism was proposed. Studies that revealed causal relationship (12, 13) coexisted with those revealed no causal relationship (14). Currently, it is still unclear whether the causality exists or not and which form of causality exists. It is SLE or drugs used to treat SLE cause thyroid diseases, or it is actually thyroid diseases or drugs used to treat thyroid diseases cause SLE.

Our study performed bidirectional two-sample univariable MR analysis and multivariable MR analysis. In univariable analysis, causal relationships of SLE on hypothyroidism, hyperthyroidism on SLE and hypothyroidism on SLE and no causal relationship of SLE on hyperthyroidism were revealed. The stability and reliability of these results were verified by sensitivity analysis. However, in the process of univariable MR analysis, an important thing was found that some SNPs were strongly correlated with both hyperthyroidism and hypothyroidism. Therefore, a multivariable MR analysis was conducted to take both hyperthyroidism and hypothyroidism as exposure. Multivariable analysis did not provide evidence to support the inverse univariable analysis results that hyperthyroidism and hypothyroidism were causally associated with SLE. Actually, causal relationship revealed by univariable MR analysis was not completely reliable. Multivariable MR analysis should be more recommended when analyzing causality. Anyway, our study revealed SLE was one cause of hypothyroidism. The greatest significance of our study was that it could alert physicians and patients to follow up thyroid function closely after the diagnosis of SLE, so as to diagnose and treat thyroid diseases as early as possible and reduce the damage of the body.

There were some obvious advantages in our study. Firstly, our study was the first research to analyze the causal relationship between SLE and hyperthyroidism and hypothyroidism by using bidirectional two-sample univariable and multivariable Mendelian randomization. In addition, our study population was large, including 402,195 samples and 39,831,813 single-nucleotide polymorphisms (SNPs). The exposure and outcome datasets obtained from different databases, which reduced the interference of sample overlap (33). Valid IVs used in our study were SNPs with strong association (*P* < 5*10^-8^) and high intensity (*F*-statistics > 10). Thus, the exposure and outcome samples in this study were more comparable and our conclusions were more convincing. Moreover, our sensitivity analysis was comprehensive, including three methods: heterogeneity test, pleiotropy test and leave-one-out test. More importantly, based on the findings during univariable MR analysis process, we performed multivariable analysis and did not accept the results of univariable analysis unceremoniously.

However, there were also several shortcomings in our study. Obviously, our study population was Europeans, not the population worldwide, so our conclusions could not be widely applied globally. The hyperthyroidism GWAS and hypothyroidism GWAS datasets included both males and females but the gender in the SLE GWAS dataset was unclear. However, it was well known that SLE affected far more women than men, with a maximum ratio of about 9:1 (1). Therefore, we believed that this weakness could not significantly change our results. Similarly, our study was not applicable for subgroup analysis based on gender. Also, SLE GWAS dataset obtained in our study was not latest published, but we believed the 14,267 samples and 7,071,163 SNPs were large enough and the 45 strongly correlated SNPs extracted from SLE GWAS were sufficient for our Mendelian analysis. Apart from this, the conclusions that we obtained from the large sample could not be used to examine the small sample effect. In addition, after revealing no causal relationship of hyperthyroidism and hypothyroidism on SLE using multivariable MR, we did not further analyze other potential exposures in the course of SLE.

In conclusion, our univariable and multivariable MR study investigated the controversial relationship between systemic lupus erythematosus and hyperthyroidism or hypothyroidism, and revealed that systemic lupus erythematosus was causally associated with hypothyroidism, but not causally associated with hyperthyroidism. Our study also implied that there might be other potential exposures in the progression of hyperthyroidism or hypothyroidism to SLE.

## Data availability statement

The original contributions presented in the study are included in the article/**Supplementary Material**. Further inquiries can be directed to the corresponding author.

## Author contributions

QQ contributed to data acquisition and analysis, as well as manuscript drafting and revision. LZ, AR and WL contributed to the data analysis and manuscript revision. RM and QP contributed to the manuscript revision. SL contributed to the supervision of the research and the revision of the manuscript. All authors reviewed and approved the paper for publication.
