# Supplementary material for: Systemic lupus erythematosus is causally associated with hypothyroidism, but not hyperthyroidism: A Mendelian randomization study
Source: Front Immunol. 2023 Feb 13;14:1125415. doi: 10.3389/fimmu.2023.1125415 (PMC9968792; doi:10.3389/fimmu.2023.1125415)
Supplement: Supplementary file 1 [file DataSheet_1.docx]

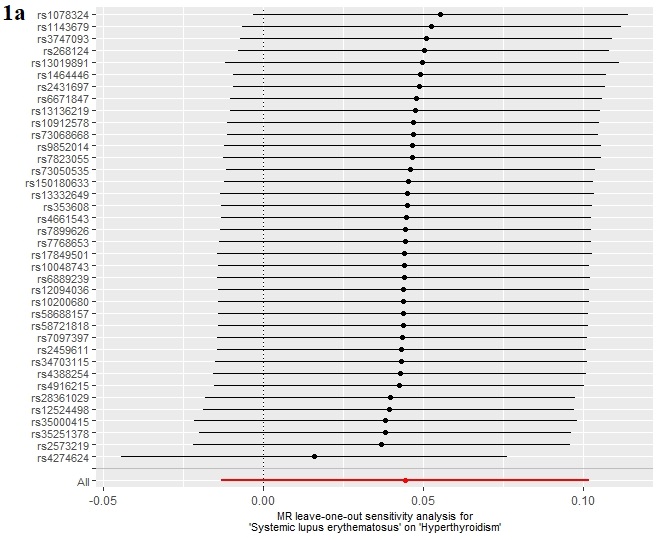


**Supplementary Figure 1a：Leave-one-out test plot of causal effect estimates by MRE-IVW for SLE on hyperthyroidism, with all 38 valid instrumental variables.**

SLE, systemic lupus erythematosus. MRE-IVW, multiplicative random effects-inverse variance weighted.


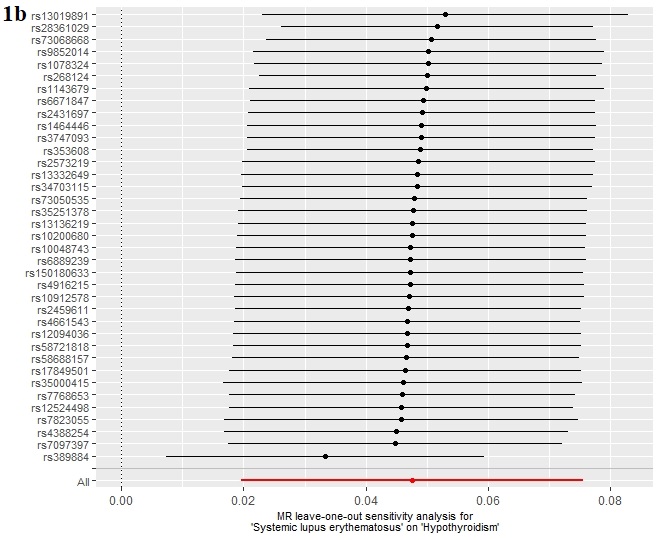


**Supplementary Figure 1b: Leave-one-out test plot of causal effect estimates by MRE-IVW for SLE on hypothyroidism, with all 37 valid instrumental variables.**

SLE, systemic lupus erythematosus. MRE-IVW, multiplicative random effects-inverse variance weighted.


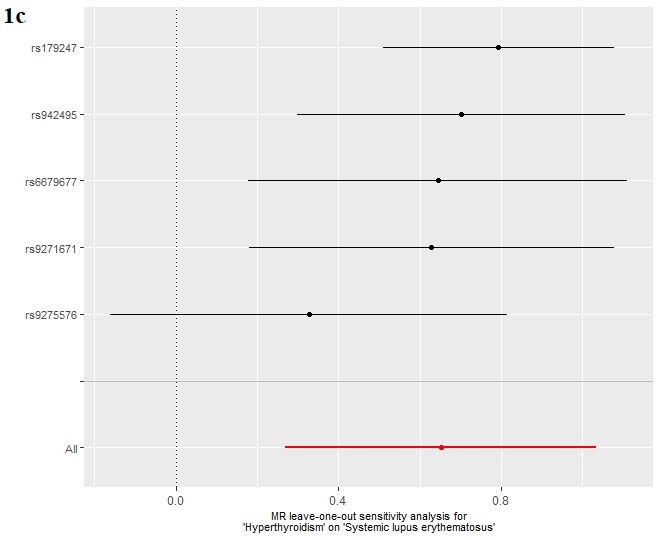


**Supplementary Figure 1c：Leave-one-out test plot of causal effect estimates by MRE-IVW for hyperthyroidism on SLE, with all 5 valid instrumental variables.**

SLE, systemic lupus erythematosus. MRE-IVW, multiplicative random effects-inverse variance weighted.


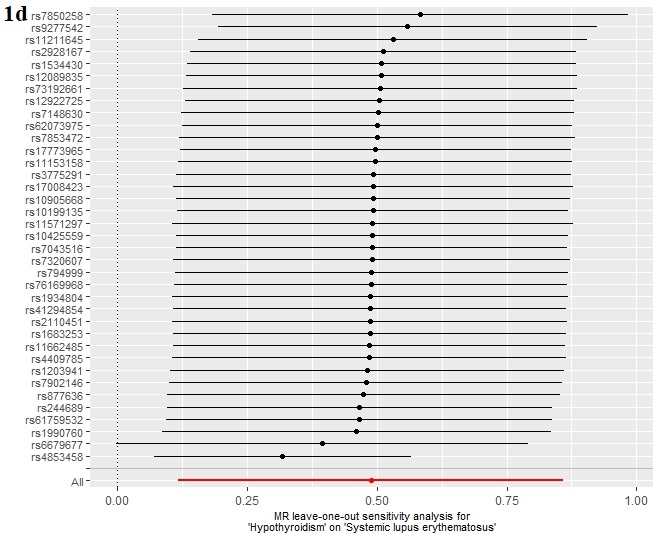


**Supplementary Figure 1d: Leave-one-out test plot of causal effect estimates by MRE-IVW for hypothyroidism on SLE, with all 37 valid instrumental variables.**

SLE, systemic lupus erythematosus. MRE-IVW, multiplicative random effects-inverse variance weighted.


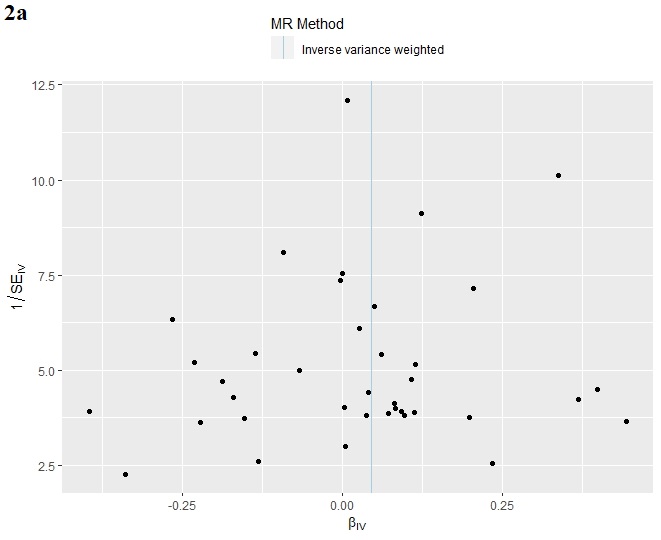


**Supplementary Figure 2a：Funnel plot of causal effect estimates for SLE on hyperthyroidism, with all 38 valid instrumental variables.**

SLE, systemic lupus erythematosus. IV, instrumental variable.


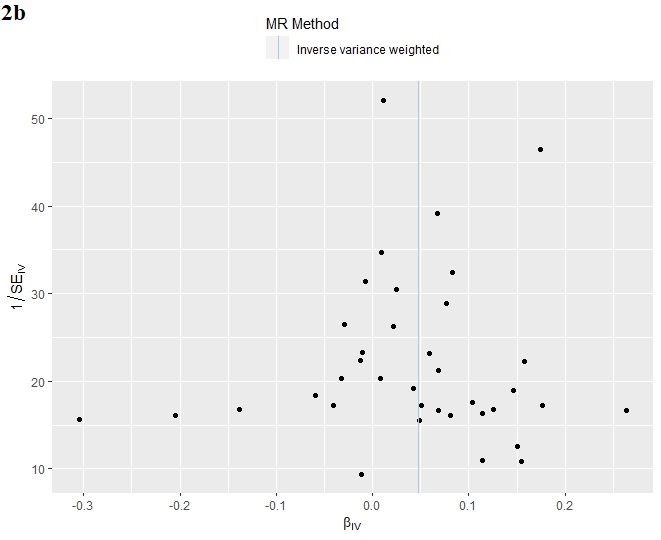


**Supplementary Figure 2b: Funnel plot of causal effect estimates for SLE on hypothyroidism, with all 37 valid instrumental variables.**

SLE, systemic lupus erythematosus. IV, instrumental variable.


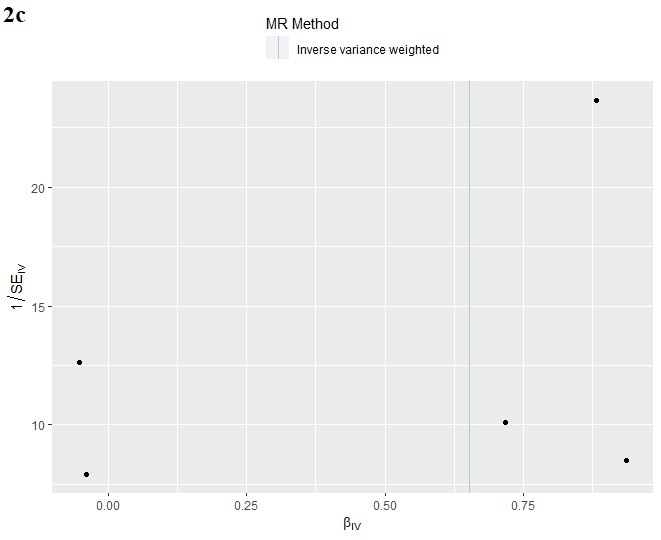


**Supplementary Figure 2c：Funnel plot of causal effect estimates for hyperthyroidism on SLE, with all 5 valid instrumental variables.**

SLE, systemic lupus erythematosus. IV, instrumental variable.


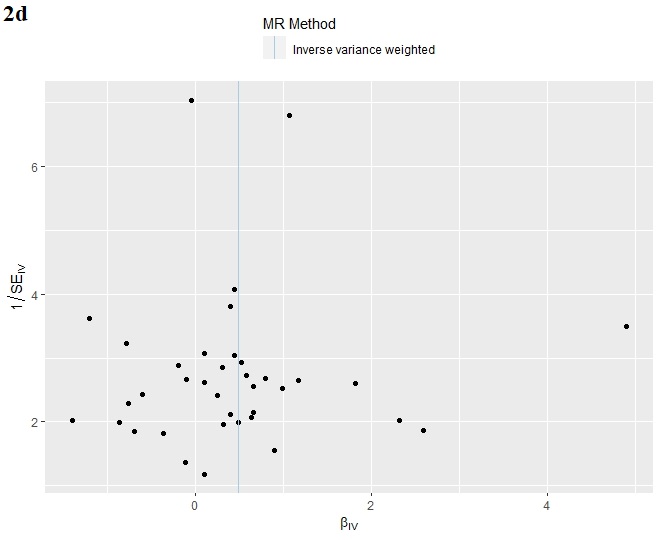


**Supplementary Figure 2d: Funnel plot of causal effect estimates for hypothyroidism on SLE, with all 37 valid instrumental variables.**

SLE, systemic lupus erythematosus. IV, instrumental variable.


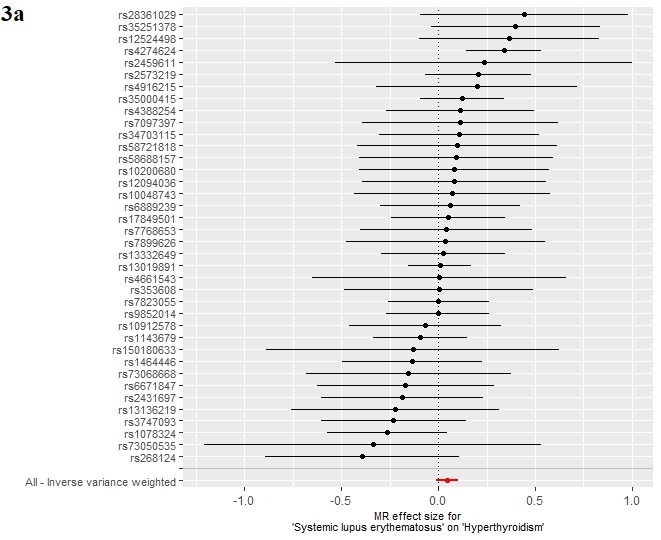


**Supplementary Figure 3a：Forest plot of causal effect estimates for SLE on hyperthyroidism, with all 38 valid instrumental variables.**

SLE, systemic lupus erythematosus.


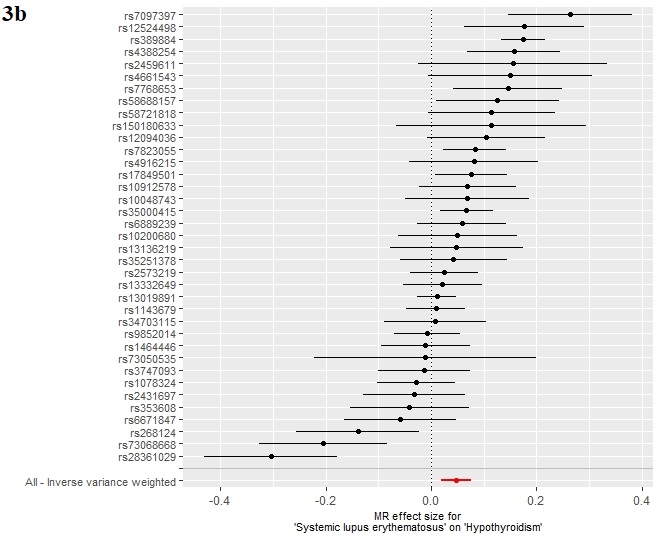


**Supplementary Figure 3b: Forest plot of causal effect estimates for SLE on hypothyroidism, with all 37 valid instrumental variables.**

SLE, systemic lupus erythematosus.


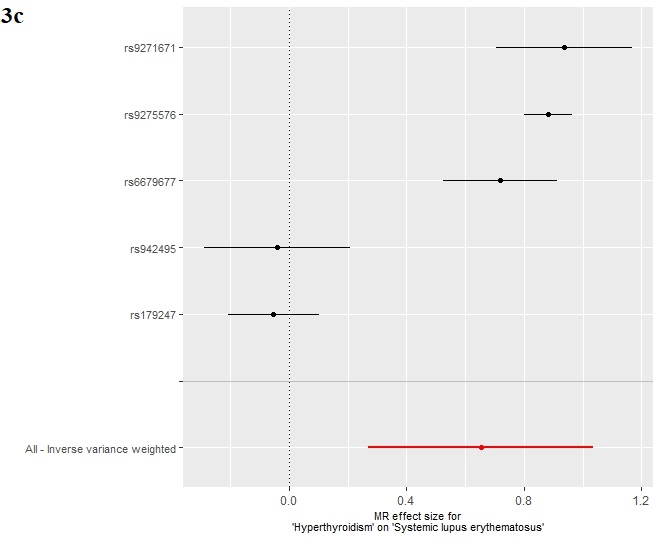


**Supplementary Figure 3c：Forest plot of causal effect estimates for hyperthyroidism on SLE, with all 5 valid instrumental variables.**

SLE, systemic lupus erythematosus.


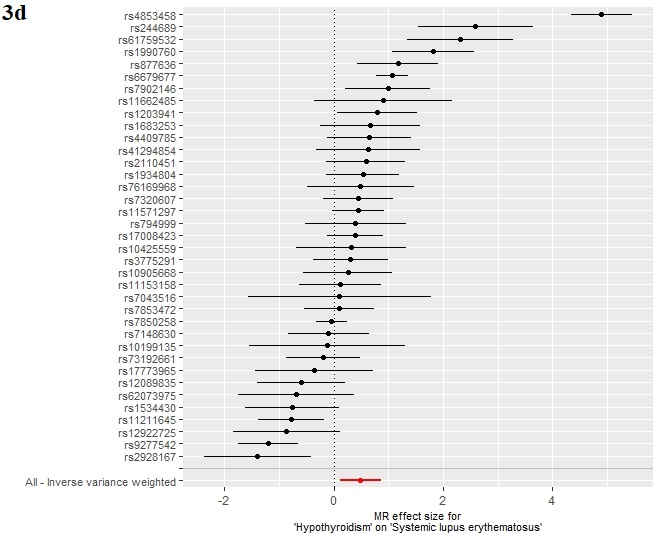


**Supplementary Figure 3d: Forest plot of causal effect estimates for hypothyroidism on SLE, with all 37 valid instrumental variables.**

SLE, systemic lupus erythematosus.
